# The Novelty of Inhalable Medications: Interest, Use, and Impact of Inhalant Medications in Low-Resource Settings

**DOI:** 10.24248/EAHRJ-D-17-00080

**Published:** 2018-04-01

**Authors:** Emily R MacGuire, Katelin Moran, Bonnie J Hepler, Doris J Rouse

**Affiliations:** 1 RTI International. Research Triangle Park, NC, USA

## Abstract

**Introduction:**

Postpartum haemorrhage (PPH) claims more than 100,000 maternal lives annually worldwide, most of them in low-resource settings. To address morbidity and mortality from PPH, the global health community is exploring novel drug formulations, such as inhalable medicine, to improve treatment availability and use, especially in community settings with limited access to skilled birth attendants. A major limitation in the ability to prevent or treat PPH in resource-limited settings is that the most effective medications for prevention and treatment are injectables, which require administration by skilled birth attendants.

**Methodology:**

We conducted formative research, including online surveys and in-person interviews, with a range of providers across a variety of health-care settings in Guatemala, Indonesia, Kenya, and Nigeria, to better understand the standard of care for mothers and newborns in low-resource settings, including care practices related to PPH.

**Results:**

It is estimated that up to 40% of PPH deaths could be averted if an inhalable prevention and treatment were available. However, survey and interview respondents noted a desire for more intravenous and oral medicinal formulations over inhalable formulations.

**Discussion/Conclusion:**

Lack of knowledge and use of inhalable medicines among these health workers illuminates key challenges to introducing novel formulations in low-resource settings.

## INTRODUCTION

### Postpartum Haemorrhage in Context in Low- and Middle-Income Countries

Each year, postpartum haemorrhage (PPH) claims the lives of more than 100,000 mothers worldwide.^1^ Most of these maternal deaths occur in low-resource settings at the community level, where it is challenging to prevent and treat PPH due to the lack of adequate health-care infrastructure, long distances from care, and technologies not available in or not appropriate for low-resource settings.^2^ In a World Health Organization (WHO) systematic analysis on maternal mortality, regional PPH-related mortality was estimated at 13.3% (n=9,200) in Latin and Central America, 15.2% (n=200,000) in sub-Saharan Africa, and 23.1% (n=34,000) in Southeastern Asia.^3^

Most low- and middle-income countries (LMICs) follow WHO recommendations for PPH prevention and treatment guidelines. Intravenous or intramuscular oxytocin for prevention and intravenous oxytocin for treatment of PPH is the WHO-recommended utero-tonic.^4^ In cases where oxytocin is not available, misoprostol or injectable uterotonics are recommended as alternative treatments.^4^

### Initial Challenges: Unmet Need and Barriers

In its current formulation, oxytocin is included in treatment guidelines and is considered the standard of care for PPH; however, it is often not available in resource-limited settings where injection is not possible.^5^ A study by Smith et al surveyed 37 countries about the inclusion of oxytocin on their Essential Medicines List (EML) and distribution of oxytocin.^5^ Of the countries surveyed, 4 reported not having oxytocin regularly available in facilities and only Equatorial Guinea did not include oxytocin on its EML for PPH prevention and treatment.^5^ Unfortunately, despite the availability of oxytocin to health-care providers within a country, it is often not available or cannot be used appropriately in community settings where many births occur. This unmet need persists throughout sub-Saharan Africa, where only 50% of births are facility-based.^6^

In low-resource settings, providing care for women suffering from PPH can be challenging for myriad reasons. including numerous barriers to care-seeking and limited skills of providers. For women in LMICs, obstacles to care-seeking and the timely effective treatment of PPH include the decision to deliver at home instead of a health-care facility.^7^

### Formulations of Oxytocin Under Development

Because oxytocin requires a refrigerated environment and administration by trained providers, women who give birth in community settings are unlikely to receive the medication when they need it.^8^ This unmet need for PPH treatment has spawned innovative approaches to bring oxytocin to communities in a form that can be administered by the local health workforce, many of whom lack formal training. The United Nations Commission on Life-Saving Commodities for Women and Children reports several examples of potential product innovations, including temperature monitoring devices for oxytocin packaging; heat-stable formulations; pre-loaded, single-use injection devices for use by lower-skilled cadres of health-care providers; and non-parenteral inhalation/intranasal spray-dried (dry powder) formulations.^9^

The inhalant form of oxytocin that is under development is described as an innovative, heat-stable, and low-cost form of oxytocin that could help manage PPH treatment in resource-poor settings.^10,11^ Such a novel approach is needed for women who do not deliver in facilities that have appropriate interventions for PPH.

## METHODS

We conducted a small-scale, mixed-methods study of maternal and neonatal health care in 4 LMICs—Guatemala, Indonesia, Kenya, and Nigeria. The mixed-methods approach included a comprehensive literature review, an online quantitative survey and qualitative interviews with health-care providers from the target countries, and mathematical modelling to assess the impact of maternal and neonatal health interventions. For the purposes of this paper, we will focus on the availability, practices, and preferences regarding PPH prevention and treatment that we explored in the study.

Data sources, including national demographic and health surveys, grey literature, and peer-reviewed journals, were derived from a literature review in PubMed using a publication date range of 2005 to 2016, with search terms: ‘postpartum haemorrhage’, ‘inhalant medication’, ‘inhalable medication’, and ‘oxytocin’ combined with ‘low-resource’ and the countries included in the study. We used a snowball methodology to review additional data sources cited by articles in our initial review.

The original survey was developed in English and then translated into Spanish and Bahasa Indonesia for the Guatemala and Indonesia sites, respectively. The survey was piloted with 1 or 2 practitioners in each country to ensure the appropriate translation of each question and the functionality of the survey platform. The feedback provided from these pilot surveys was incorporated into the final version of each country survey. Across the 4 sites, health-care providers were identified for the online survey through convenience sampling; respondents represented the variety of providers – doctors, nurses, midwives, and traditional birth attendants – who care for pregnant women. By country, the final number of respondents was: Guatemala (n=11), Indonesia (n=11), Kenya (n=10), and Nigeria (n=10). The number of respondents for this small-scale study was determined as a number both sufficient to inform a product development strategy and practical within study constraints. For geographic context, the respondents from Guatemala represent semi-urban health facilities near Guatemala City, while respondents from Kenya are from rural western Kenya. The Nigerian practitioners are from the Kano area, representing a semi-urban region, and the Indonesian respondents are also mainly from an urban area. We sought a broad range of feedback from across the health provider spectrum, as noted in the characteristics of the survey respondents provided in **Table 1**.

**TABLE 1.** Characteristics of Survey Respondents

Country	Profession	Average Years of Experience	Type of Facility
Guatemala (n=11)	Doctor (5)	16–20 years	Public hospital (4)
	Nurse (4)		Health clinic (2)
	Traditional birth attendant (2)		Health area (2)
			Patient's home/village setting (2)
Indonesia (n=11)	Doctor (5)	16–20 years	Public hospital (1)
	Nurse (3)		Health clinic (10)
	Midwife (3)		
Kenya (n=10)	Doctor (4)	6–10 years	Public hospital (9)
	Nurse (5)		Health clinic (1)
	Midwife (1)		
Nigeria (n=10)	Doctor (1)	10 years	Public hospital (7)
	Nurse (3)		Health clinic (2)
	Midwife (1)		Tertiary institution (1)
	Community health worker (5)		

Prior to launching the qualitative interviews, the interview questionnaire, which included scripted questions, was sent to all sites for translation, feedback, and clarification. Each qualitative interview was conducted in person with individual health-care providers from each country. Many of the qualitative interview participants also contributed as survey respondents, including 5 providers from Guatemala, 1 provider from Kenya, and 4 providers from Nigeria. The final number of interview respondents by country was: Guatemala (n=5), Indonesia (n=4), Kenya (n=4), and Nigeria (n=5). **Table 2** provides the characteristics of the interview respondents. The data collected during the interviews were analysed through content analysis.

**TABLE 2.** Characteristics of Interview Participants

Country	Profession	Average Years of Experience	Type of Facility
Guatemala (n=5)	Doctor (3)	10 years	Hospital (4)
	Obstetrician-gynaecologist (1)		Health centre (1)
	Nurse (1)		
Indonesia (n=4)	Doctor (2)	15 years	Health centre (4)
	Midwife (2)		
Kenya (n=4)	Doctor (2)	9 years	Hospital (4)
	Nurse/midwife (1)		
	Clinical officer (1)		
Nigeria (n=5)	Doctor (1)	14 years	Hospital (2)
	Nurse/midwife (2)		Public health facility (2)
	Nurse (1)		
	Community health extension worker (1)		

The PPH portion of the survey focused on questions related to available treatments for the third stage of labour, preferred formulations, refrigeration availability in facilities, and the use of inhaled delivery of medications for any cause (see Appendix A for the full set of PPH-related survey questions). Further, through qualitative interviews, we explored the barriers associated with inhalable medicine across a variety of health-care providers. The PPH-related interview question is also included in Appendix A.

As part of the PPH treatment analysis we modelled the potential impact of inhalant oxytocin using the Maternal and Neonatal Directed Assessments of Technology (MANDATE) model, an interactive web-based decision-tree mathematical model. MANDATE, developed by RTI with support from the Bill and Melinda Gates Foundation, informs the development of appropriate and effective technologies to improve maternal and neonatal health care in low-resource settings (www.mnhtech.org). Using this baseline model, researchers can evaluate theoretical scenarios that improve baseline data across prevention, diagnostic, transfer, and/or treatment data, resulting in lives saved across care settings. Baseline assumptions for sub-Saharan Africa are outlined in **Table 3**. The inputs of the MANDATE model and the current estimates of penetration (availability of an intervention), utilization (appropriate use of an intervention), and efficacy (ability of an intervention to successfully prevent, treat, or diagnose a condition) can be used to run scenarios to examine the impact of the improvement of an intervention or a package of interventions in 1 or more care settings.^12^

**TABLE 3.** Baseline Assumptions for Model

Sub-Saharan Africa 2020 Baseline Assumptions				
Intervention	Metric	Home	Clinic	Hospital
**Prevention Interventions**				
Uterine massage to prevent PPH	Penetration	50%	95%	100%
	Utilization	35%	40%	45%
	Efficacy	40%	40%	40%
Oxytocin to prevent PPH	Penetration	0%	60%	80%
	Utilization	0%	65%	65%
	Efficacy	50%	50%	50%
Misoprostol to prevent PPH	Penetration	5%	15%	20%
	Utilization	55%	65%	65%
	Efficacy	43%	43%	43%
Drape (to determine blood loss)	Penetration	0%	0%	1%
	Utilization	0%	0%	10%
	Efficacy	92%	92%	92%
Recognize PPH	Penetration	50%	0%	0%
	Utilization	60%	0%	0%
	Efficacy	60%	60%	60%
Clinical diagnosis of PPH	Penetration	0%	85%	90%
	Utilization	0%	80%	90%
	Efficacy	0%	70%	70%
Recognize retained placenta	Penetration	50%	0%	0%
	Utilization	15%	0%	0%
	Efficacy	70%	70%	70%
Clinical diagnosis of a etiology of PPH (lacerations, atonic uterus, retained placenta)	Penetration	0%	85%	90%
	Utilization	0%	65%	80%
	Efficacy	0%	85%	85%
Ultrasound for retained placenta	Penetration	0%	0%	25%
	Utilization	0%	0%	10%
	Efficacy	85%	85%	85%
Manual removal of retained placenta	Penetration	30%	75%	99%
	Utilization	30%	40%	45%
	Efficacy	70%	70%	70%
D&C, manual removal	Penetration	0%	30%	70%
	Utilization	0%	30%	40%
	Efficacy	90%	90%	90%
Surgery for retained placenta	Penetration	0%	0%	75%
	Utilization	0%	0%	50%
	Efficacy	92%	92%	92%
Recognize lacerations	Penetration	50%	0%	0%
	Utilization	15%	0%	0%
	Efficacy	70%	70%	70%
**Treatment Interventions**				
Clinical diagnosis of a etiology of PPH (lacerations, atonic uterus, retained placenta)	Penetration	0%	85%	90%
	Utilization	0%	65%	80%
	Efficacy	0%	85%	85%
Suturing	Penetration	30%	80%	95%
	Utilization	50%	70%	95%
	Efficacy	90%	90%	90%
Surgery – lacerations	Penetration	0%	0%	75%
	Utilization	0%	0%	50%
	Efficacy	92%	92%	92%
Recognize atonic uterus	Penetration	50%	0%	0%
	Utilization	15%	0%	0%
	Efficacy	15%	15%	15%
Clinical diagnosis of a etiology of PPH (lacerations, atonic uterus, retained placenta)	Penetration	0%	85%	90%
	Utilization	0%	65%	80%
	Efficacy	0%	85%	85%
Ultrasound – atonic uterus	Penetration	0%	0%	25%
	Utilization	0%	0%	10%
	Efficacy	85%	85%	85%
Uterine massage to treat PPH	Penetration	50%	95%	100%
	Utilization	35%	60%	80%
	Efficacy	40%	40%	40%
Oxytocin to Treat PPH	Penetration	0%	60%	80%
	Utilization	0%	65%	65%
	Efficacy	50%	50%	50%
Misoprostol to Treat PPH	Penetration	5%	15%	20%
	Utilization	55%	65%	65%
	Efficacy	43%	43%	43%
Balloon tamponade	Penetration	0%	0%	0%
	Utilization	0%	0%	0%
	Efficacy	0%	0%	0%
Surgery – atonic uterus	Penetration	0%	0%	75%
	Utilization	0%	0%	50%
	Efficacy	92%	92%	92%
Recognize severe haemorrhage	Penetration	50%	0%	0%
	Utilization	70%	0%	0%
	Efficacy	80%	0%	0%
Clinical diagnosis of severe haemorrhage	Penetration	0%	85%	90%
	Utilization	0%	90%	99%
	Efficacy	0%	95%	95%
Fluid resuscitation	Penetration	0%	50%	99%
	Utilization	0%	70%	80%
	Efficacy	5%	5%	5%
Blood replacement and transfusion	Penetration	0%	5%	45%
	Utilization	0%	50%	80%
	Efficacy	95%	95%	95%
**Impact of Transfer**				
Transfer with diagnosis of PPH	Home to clinic		30%	
	Home to hospital		15%	
	Clinic to hospital		35%	
Transfer with diagnosis of retained placenta	Home to clinic		30%	
	Home to hospital		15%	
	Clinic to hospital		35%	
Transfer with diagnosis of lacerations	Home to clinic		30%	
	Home to hospital		15%	
	Clinic to hospital		35%	
Transfer with diagnosis of atonic uterus	Home to clinic		30%	
	Home to hospital		15%	
	Clinic to hospital		35%	
Transfer with diagnosis of severe haemorrhage	Home to clinic		30%	
	Home to hospital		20%	
	Clinic to hospital		40%	

Abbreviations: D&C, dilation and curettage; PPH, postpartum haemorrhage.

## RESULTS

### Modelling Potential Impact

We ran a series of MANDATE scenarios for sub-Saharan Africa to assess the number of maternal lives saved through the scale up of an inhalable form of oxytocin for PPH prevention and treatment. The results are summarized in **Table 4**. The first scenario shows that people – across all settings – who do not currently have access to (penetration) or use (utilization) oxytocin will now have increased access to and use of oxytocin. In this first scenario, both penetration and utilization are increased to ensure that 50% of people who do not have access to oxytocin get access to the medication and 50% of people who do not use oxytocin will now be able to access and use the medication. The second scenario shows near perfect access and use of oxytocin across all settings, which means that penetration (availability) and utilization (use) both raised to 99%. MANDATE estimates approximately 40% of all maternal deaths from PPH could be prevented through the universal use of an inhalable form of oxytocin.

**TABLE 4.** MANDATE Model: Estimated Number of Maternal Deaths and Lives Saved With Improvement of Postpartum Haemorrhage Prevention and Treatment Through an Inhalable Oxytocin, Sub-Saharan Africa, 2020

Scenario		Maternal Deaths (n)	Maternal Lives Saved From Current Level (n)	Maternal Deaths Prevented From Current Level (%)
Number	Scenario			
1.	Current levels of prevention and treatment	54,290	n/a	n/a
2.	Moderate coverage (50%) inhalable oxytocin prevention and treatment: all settings	45,600	8,690	16
3.	Near perfect coverage (95%) of inhalable oxytocin prevention and treatment: all settings	33,790	20,500	38

### Refrigeration Capabilities

In LMIC health-care settings, drugs that are not heat stable – including current formulations of oxytocin – degrade and, thus, require refrigeration.^8,13^

It is important to note, across all the regions that we surveyed, only a third or less of all clinics and at least half of the health centres in all regions had the ability to store medicine in refrigerated conditions. The majority of hospitals across all regions had the ability to refrigerate medicine; however, in Indonesia, health-care providers stated that hospitals were less likely to be able to refrigerate medicine than health centres. The responses are summarized in **Table 5**.

**TABLE 5.** Refrigeration Capabilities of Health Care Settings

	Clinic	Health Centre	Hospital
Guatemala (n=11)	18%	55%	91%
Indonesia (n=11)	18%	82%	64%
Kenya (n=10)	20%	70%	100%
Nigeria (n=10)	20%	50%	80%

Note: In this study, clinic refers to village/community-based health care; health centre and hospital refer to urban, regionally based care.

The lack of proper storage facilities and ability to maintain the cold chain, especially at the community level, underscores the necessity for innovative formulations that can work within current health systems and contexts.^14^

### Formulation Preferences

Despite the potential impact of a universally heat-stable inhalable version of oxytocin that may allow greater access and ease of use for less-skilled health workers in low-resource settings, health-care providers surveyed in the 4 LMICs reported little to no use of any inhalable medicine. Across all sites and all health-care providers, no respondent reported a preference for using an inhalable form of PPH medicine. In all country sites examined, the majority of health-care providers noted that either inhaled delivery medicines were not used or that they were unaware of these formulations being used.

Across all sites, intravenous formulations were a preferred formulation for PPH (see **Table 6** for a summary of preferred formulations). Health-care providers also preferred oral formulations in Guatemala and Indonesia; intramuscular formulations in Guatemala, Kenya, and Nigeria; and suppositories in Nigeria. The responses are summarized in **Table 6**.

**TABLE 6.** Preferred Formulation for Postpartum Haemorrhage Medications

	Inhaled	Intravenous	Oral	Transdermal	Sublingual	Suppositories	Intramuscular	Other	Don't Know
Guatemala (n=11)	0%	64%	55%	0%	9%	18%	40%	0%	9%
Indonesia (n=11)	0%	64%	64%	0%	9%	27%	27%	0%	0%
Kenya (n=10)	0%	100%	0%	0%	10%	10%	60%	0%	0%
Nigeria (n=9)	0%	44%	11%	0%	33%	33%	33%	0%	0%

Note: Answers are not mutually exclusive.

Of note, innovative formulations, including inhaled formulations, were not selected by the survey participants. Instead, the formulations they regularly used seem to be the formulations they prefer. Across all settings, efficacy and availability were cited as the primary reasons for their preferred formulation. Further, when asked about their desired PPH formulation, providers expressed the desire for more intravenous and oral medicines over inhalable formulations. These responses are summarized in **Table 7**.

**TABLE 7.** Desired Formulation of Postpartum Haemorrhage Medicine

	Inhaled	Intravenous	Oral	Transdermal	Sublingual	Suppositories	Other	Don't Know
Guatemala (n=11)	0%	27%	27%	0%	0%	9%	0%	36%
Indonesia (n=11)	0%	9%	27%	0%	0%	9%	0%	27%
Kenya (n=10)	40%	20%	20%	10%	30%	20%	0%	40%
Nigeria (n=9)	0%	33%	33%	0%	44%	22%	11%	0%

Note: Answers are not mutually exclusive.

Notably, 40% of Kenyan respondents, including 2 doctors, 1 nurse, and 1 midwife, were the only respondents across the entire study to indicate that they would desire an inhalant formulation for PPH medication.

### Qualitative Insights From Inhalable Questions

Qualitative interviews revealed that respondents had little experience and/or understanding of inhalable medicines in general. Across the countries surveyed, all health-care providers stated that the primary barriers associated with using inhaled medicine were that they have never been used before and/or were not available. For example, a respondent in Indonesia explained in response to the question ‘In your region, what barriers are associated with inhaled medicine?’ that ‘I do not know anything about inhaled medications, so far all I know is the other methods.’ The lack of knowledge about inhalable formulations and their availability are considerable barriers to the uptake of inhalable medicines. It is evident that the respondent health-care providers trust their current formulations. One respondent from Nigeria noted other barriers for inhalable medications, stating the ‘people surrounding the patient, they all have effect (of the medication) also, so that one is also a barrier too; and then I think the rule doing an inhalational form is not a fast route.’ If a new drug or formulation is introduced to the market, health-care providers will need to trust the supply chain and the impact of the medicine in order to adopt its use and be trained on the new delivery system of inhalables.

## DISCUSSION

Our research fits into the global research context outlined in the United Nations Commission on Life-Saving Commodities for Women and Children report, in which oxytocin is identified as one of 13 life-saving commodities.^9^ The report provides recommendations for actions needed to increase uptake and use of these crucial commodities.^9^

Our small study begins to address some of the challenges associated with introducing a new formulation for PPH prevention and treatment. Through our research, we identified which countries include oxytocin in their treatment guidelines and measured levels of health-care provider knowledge and use of various formulations of oxytocin. We further explored the availability of oxytocin and challenges to its use, including preferred and desired formulations.

Although paediatric tuberculosis (TB) is a different field, the challenges in introducing new formulations of medication are similar to those posed in the maternal and neonatal health field. In their review of barriers to uptake of paediatric TB treatment, Craig et al^15^ highlight the need for more qualitative data about patient, caregiver, and provider knowledge, behavior, beliefs, and attitudes regarding medicine in general and biomedical pharmaceuticals, in particular to inform the introduction of new formulations.^15^ Our study begins to fill this gap in qualitative field data to inform formulation uptake that Craig et al note by surveying a variety of health-care providers as well as the mixed-methods approach that was used for data collection. Respondents were chosen from a variety of urban and rural health-care settings, representing each level of care – from the community to the hospital level. Insights from a variety of providers, including a range of community health workers with basic training and physicians with specialty training in obstetrics and gynaecology, further strengthened the results. Moreover, respondents had various years of experience in the health-care field, providing both novice and expert practitioner perspectives. Finally, the combination of literature review, modelling, and quantitative and qualitative data collected through the surveys and interviews provides a comprehensive set of results.

### Limitations

It should be noted that the intent of this study was to provide initial insights into maternal and neonatal health care in the selected countries. The sample size was small, which may give results that do not adequately represent the larger population and may not reflect regional variations within the countries. The survey respondents may not have considered the inhalant form of oxytocin because they are unfamiliar with inhalables, in general, or they did not know an inhalable formulation of oxytocin could be available in the future. While valuable information was gleaned from this study, additional studies could examine variations among respondents and/or gather additional insights into the context of the responses from those interviewed and surveyed.

## CONCLUSION

Oxytocin is currently formulated as an injectable medicine that is not heat stable – requiring refrigeration and maintenance of the cold chain – and must be administered by trained health-care providers. A heat stable, inhalable version of oxytocin may allow greater access and ease of use, making it possible for it to be administered by less skilled health-care providers in low-resource settings. However, the health-care providers surveyed reported little to no use of any inhalable medicine. Further, providers expressed the desire for more intravenous and oral medicinal formulations over inhalable formulations. Interviews revealed that respondents had little experience and/or understanding of inhalable medicine, and therefore no interest in this formulation.

Given the low use and the lack of knowledge about inhalable medicine among the survey respondents, education around the use of an inhalable formulation would be needed to increase health-care providers' interest and self-efficacy in administering this novel treatment method once it is fully developed.

Additional research opportunities related to the selection of preferred or desired formulations could help put a new form of the gold standard PPH treatment in the hands of frontline health-care providers. Of note, 40% of Kenyan provider respondents stated that their desired formulation would be an inhalable formulation. In Kenya, the Ministry of Health is currently attempting to increase access to oxytocin.^15^ Further exploration of Kenyan provider interest in inhalant formulations is needed to understand if there is a link between the Ministry of Health's campaign and the expressed interest in inhalant treatment. Research into why an easier mechanism to deliver PPH treatment did not rise to the top of preferred or desired formulations in other locations could inform the need to create awareness and demand before an inhalant form of oxytocin is market ready.
